# Quality, Equity and Partnerships in Mixed Methods and Qualitative Research during Seven Years of Implementing the Structured Operational Research and Training Initiative in 18 Countries

**DOI:** 10.3390/tropicalmed7100305

**Published:** 2022-10-17

**Authors:** Rony Zachariah, Arpine Abrahamyan, Stefanie Rust, Pruthu Thekkur, Mohammed Khogali, Ajay M. V. Kumar, Hayk Davtyan, Srinath Satyanarayana, Hemant D. Shewade, Alexandre Delamou, Maria Zolfo, Veerle Hermans, Selma Dar Berger, Anthony Reid, Abraham Aseffa, Amol R. Dongre, Anthony D. Harries, John C. Reeder

**Affiliations:** 1 UNICEF, UNDP, World Bank, WHO, Special Programme for Research and Training in Tropical Diseases (TDR), CH-1211 Geneva, Switzerland; 2 Tuberculosis Research and Prevention Center NGO (TB-RPC), Yerevan 0014, Armenia; 3 Local Health Authority, District of Diepholz, 49356 Diepholz, Germany; 4 Centre for Operational Research, International Union against Tuberculosis and Lung Disease (The Union), 75001 Paris, France; 5 The Union South-East Asia Office, C6, Qutub Institutional Area, New Delhi 110016, India; 6 Yenepoya Medical College, Yenepoya (Deemed to Be University), Deralakatte, Mangaluru 575018, India; 7 Division of Health Systems Research, ICMR-National Institute of Epidemiology (ICMR-NIE), Chennai, Tamil Nadu 600077, India; 8 Department of Public Health, Gamal Abdel Nasser University of Conakry, Conakry 1147, Guinea; 9 Institute of Tropical Medicine, 2000 Antwerp, Belgium; 10 Médecins Sans Frontières, Operational Centre Brussels, LuxOR, 1617 Luxembourg, Luxembourg; 11 Pramukhswami Medical College (PSMC), Karamsad 388325, India; 12 Department of Clinical Research, Faculty of Infectious and Tropical Diseases, London School of Hygiene and Tropical Medicine, London WC1E 7HT, UK

**Keywords:** COREQ, operational research, SORT IT, qualitative studies, mixed-methods, universal health coverage, health system resilience

## Abstract

**Introduction**: Qualitative studies are often inadequately reported, making it difficult to judge their appropriateness for decision making in public health. We assessed the publication characteristics and quality of reporting of qualitative and mixed-method studies from the Structured Operational Research and Training Initiative (SORT IT), a global partnership for operational research capacity building. **Methods:** A cross-sectional analysis of publications to assess the qualitative component using an adapted version of the Consolidated Criteria for Reporting Qualitative Research (COREQ) checklist. **Results:** In 67 publications involving 18 countries, 32 journals and 13 public health themes, 55 were mixed-methods studies and 12 were qualitative studies. First authorship from low-and-middle-income (LMIC) countries was present in 64 (96%), LMIC last authorship in 55 (82%), and female first authorship in 30 (45%). The mean LMIC institutions represented per publication was five (range 1–11). Sixty-three (94%) publications were open access. Reporting quality was graded as ‘good’ to ‘excellent’ in 60 (89%) publications, ‘fair’ in five (8%) and ‘poor’ in two (3%). **Conclusion:** Most SORT IT publications adhered to COREQ standards, while supporting gender equity in authorship and the promotion of LMIC research leadership. SORT IT plays an important role in ensuring quality of evidence for decision making to improve public health.

## 1. Introduction

Decision makers often seek responses to questions in the real-world, such as on treatment outcomes, access to healthcare in different settings and on how to deliver services in various contexts and for different populations. Randomized controlled trials cannot answer such questions, as although they are conducted in the field, they are implemented in controlled settings with rigid inclusion and exclusion criteria which may not reflect on-the-ground realities [1,2] In the health care setting, evidence from clinical trials thus need to be applied through ‘models of delivery’ that are acceptable and effective in specific contexts and populations. Evidence from both clinical trials and operational research are important and they need to be in a continuum. Thus, the World Health Organization (WHO) increasingly relies on evidence from operational research studies for formulating its guidelines for deploying and rolling out proven interventions [3]. Operational research, which is conducted close to the supply and demand of health services, is particularly important for building the science of solutions for achieving Universal Health Coverage (UHC) [4,5]. It is defined as the search for knowledge on interventions, strategies, tools, or policies that can enhance the quality, effectiveness, or coverage of health systems [5].

Most operational research studies are quantitative in nature with cross-sectional, cohort, and case–control designs, but mixed-methods and qualitative designs are increasingly used. While quantitative studies provide information on ‘the what’ of a given problem, mixed-methods and qualitative studies shed light on ‘the why’ behind the problem. Thus while quantitative research gives us a number, qualitative research illuminates the context, which is required for decision making The latter focuses on why individuals think or act in a particular manner, using open-ended data gathering methods such as observations, key-informant/in-depth interviews or focus group discussions [6,7].

The Structured Operational Research and Training Initiative (SORT IT) is a global partnership coordinated by TDR, which is a Special Program for Research and Training in Tropical Diseases hosted at the World Health Organization and co-sponsored by UNDP, UNICEF, The World Bank, and WHO. Focused on frontline health workers in low- and middle-income countries (LMICs), it aims to build sustainable capacity to conduct operational research and use the evidence generated for decision making [5,8,9]. The goal is to make countries and institutions ‘data rich, information rich and action rich’ [8]. SORT IT currently involves 69 implementing partners including disease control programs, non-governmental organizations and academia. Covering 94 countries and over a thousand trainees, 90% of trainees have published scientific papers, with almost 70% of the research having contributed to a change in policy and/or practice [8,10].

Recognizing the need for maintaining high reporting standards in research [3,11], SORT IT incorporated the Strengthening the Reporting of Observational Studies in Epidemiology (STROBE) [12] and the Consolidated Criteria for Reporting Qualitative Research (COREQ) guidelines [13]. Both STROBE and COREQ guidelines include checklists, which help assess the completeness and quality of reporting into its program. A previous assessment of 392 observational studies from SORT IT projects in 72 countries revealed excellent reporting according to STROBE standards, the average score exceeding 85% [14]. No such assessment has been performed on mixed methods and qualitative studies.

The COREQ “checklist” is designed to assess reporting on the conduct and reporting of qualitative research including the data collection methods such as in-depth interviews and focus group discussions. Thus, can be used for assessing the completeness of reporting qualitative research [13]. The checklist incorporates the calculation of a score of reporting quality using 32 items as denominator. It is important to assess how well qualitative studies emerging from SORT IT meet the gold standard reporting requirements recommended by COREQ. High standards in ‘quality of reporting’ is important to ensure appropriateness for decision making by those who will use the research findings to influence policy and/or practice to improve public health. In particular, use of the COREQ checklist provides a structured framework that will reduce common procedural biases on issues such as: the criteria for participant selection; conduct of interviews in a manner that limits information bias (interviewer and responder bias) including who, where and how interviews were conducted.

In addition to assessing reporting quality, we feel operational research in LMICs needs to be evaluated through a wider lens, that includes LMIC involvement and leadership, equity, collaborative partnerships, and timely access to the generated evidence [15].

A PubMed search revealed a number of studies showing that completeness of reporting of qualitative studies ranged between 40–60%. However, these were restricted to specific themes, such as dentistry, nursing, organ transplant, and smoking practices [16,17,18,19,20], or to specific countries [21]. No study has assessed the reporting of qualitative studies stemming from a research capacity building initiative focused on LMICs and covering a spectrum of public health domains. This study is important to both operational research scientists and those from academia who are involved with research training initiatives. It will help to improve understanding on how to use the COREQ checklist, especially by those involved with similar research capacity building initiatives. It will also provide a useful baseline for future comparisons while the SORT IT brand is being franchised to and independently implemented by 69 partner institutions.

In SORT IT publications that involved a qualitative component (qualitative and mixed-methods studies), we thus assessed: (a) LMIC leadership, gender equity and collaborative partnerships in authorship, (b) timely access to publications, and (c) the quality of reporting of the qualitative components.

## 2. Materials and Methods

### 2.1. Study Design

A cross-sectional analysis of the qualitative component of publications that involved qualitative or mixed methods study designs from SORT IT courses in Asia, Africa, and Europe.

### 2.2. Study Inclusion and Period

The study included all qualitative research publications that were initiated and completed between January 2015 (when SORT IT started teaching qualitative research methods) and July 2021 (Supplementary File S1). Only the qualitative components of mixed methods studies were assessed. The study was conducted between November 2021 and July 2022.

### 2.3. The SORT IT Training Model

The core aspects of the SORT IT training model have been described before [14]. In brief, front-line health workers and decision makers acquire the theoretical and practical skills for conducting operational research. There are four modules that run over 8–12 months. Each module lasts 6–7 days: module 1 focuses on the development of a operational research protocol; module 2 on efficient data capture and analysis; module 3 on manuscript writing for peer-reviewed scientific publications; and module 4 on effective communication for improved research uptake. The modules involved lectures on various aspects of mixed-methods and qualitative research, including analytic methods. To progress from one module to the next, participants must achieve milestones. During the modules, participants work in groups with experienced mentors, who provide hands-on support from the protocol stage to the publication and on effective communication of research findings.

SORT IT also has an integrated system for training of trainers: in addition to the front-line health worker (who is the principal investigator), outstanding alumni are brought in and groomed as future mentors. After completion of the training, participants are followed-up for 18 months to assess if the acquired skills are being used and whether there is impact of the research on policy and/or practice.

### 2.4. LMIC Leadership, Gender Equity and Collaborative Partnerships

Publication themes, order of LMIC authorship and numbers and types of institutional affiliations, and if these institutions were from a high-income country (HIC) or LMIC were sourced from the title page of each published paper. ‘Gender equity’ was assessed as the proportion of publications that had a female first author and this information was sourced from the SORT IT database. For ‘collaborative partnerships’, we determined the mean number of HIC and LMIC institutions represented on each publication.

### 2.5. Timely Access to Published Evidence

The time taken to publish was the interval between the date of manuscript submission to the date of publication in a peer-reviewed journal. Date of submission was sourced from the SORT IT database and date of publication from the title page of the published paper.

Access type of journal, open or subscription-based was taken from the journal websites. Total number of downloads per paper was obtained from the journal website where available.

### 2.6. The Adapted COREQ Checklist

The standard COREQ checklist includes 32 items [13] which are grouped into three domains: (1) research team and reflexivity which includes eight items on the details of the researcher and relationship with study participants; (2) study design which includes 15 items that identify the theoretical framework, participant selection process, data collection and study setting; and (3) data analysis and reporting which includes nine items. We adapted this checklist by including three additional items: (a) local relevance of the research question; (b) presence of local ethics approval; and (c) presence of international ethics approval. Thus, our modified checklist included 35 items (including instructions for use) as shown in Supplementary File S2.

### 2.7. Assessment of Quality of Reporting Using the Adapted COREQ Checklist

The adapted checklist was piloted on a sample of ten publications. Two reviewers experienced in operational research and familiar with COREQ, reviewed and scored each of the ten articles independently. To ensure common understanding of use of the checklist and the reliability of assessments between the two reviewers, the scores were compared and cross-validated for each publication. Any disagreement in reported items was reviewed and discussed with a third senior reviewer. The rest of the papers were assessed by the primary reviewer and a random validation check of 10% of all papers was carried out by the second reviewer.

Each of the 35 items in our study received a score of ‘1′ if reported, and ‘0′ if not reported. For items that had two subcomponents, a score of 0.5 was given for each subcomponent reported. For each paper, a reporting score was calculated by dividing the number of adequately reported items (the numerator) by the total applicable items in the modified checklist (the denominator) and this was expressed as a percentage. The denominator could thus vary and be less than 35. The percentage scores for quality of reporting were graded as follows: Poor: <50%; Fair: 50–59%; Good: 60–69%; Very good: 70–79%; Excellent: >80%. The cutoff points of <50%, 50–70% and >70% were adapted from a similar grading used by Sandra Walsh et al. [20]. For the purposes of this study, quality of reporting of mixed methods studies focused on the qualitative component only.

### 2.8. Data Variables, Sources, Analysis and Statistics

Data variables included journal characteristics (journal identification; type of publication; publication themes; journal name; journal impact factor), publication characteristics including institutional affiliation of authors; institution based in a HIC or LMIC, gender of the first author, date of first submission to a journal, date of publication, access type of journal (open or subscription-based access), total number of downloads per paper, and COREQ reporting score.

The SORT IT monitoring database (Microsoft Excel 2010, version 14, USA), the title page of the published paper and the journal website were used to collect data related to the study objectives. A new database (Microsoft Excel) was created for the analysis. Data were analyzed using descriptive statistics, and the results were reported using numbers and proportions. Time to publication was presented in months using median and interquartile range. The censor date for article download metrics was 15 May 2022.

## 3. Results

### 3.1. LMIC Leadership, Gender Equity and Collaborative Partnerships

A total of 67 publications from 18 countries in 32 journals and covering 13 public health themes were included (Table 1). There were 55 mixed-methods and 12 qualitative studies. Sixty-four (96%) publications had a first author and 55 (82%) had a last author from LMICs, and in 30 (45%) publications, the first author was female. The mean number of LMIC institutions represented per publication was five (range 1–11), and for HICs this was one (range 0–5).

### 3.2. Timely Access to Publications and Downloads

The median (Interquartile range) time to publication was 14 (10–22) months. Sixty-three (94%) publications were published in open access journals. Seventeen (53%) of 32 journals where manuscripts were submitted had word count limits some as low as 2500 words for an original article. Of 19 (28%) publications for which article download metrics were available, there were 12 989 downloads (an average of 684 downloads per paper).

### 3.3. Quality of Reporting

Table 2 shows quality of reporting of publications in line with an adapted COREQ checklist. Of all publications (N-67), reporting quality was ‘good’ to ‘excellent’ in 60 (89%), ‘fair’ in five (8%) and ‘poor’ in two (3%).

Table 3 shows the percentage by item in the adapted COREQ check list which were reported, not reported and not applicable.

17 of 35 items were reported in >90% of papers. Of three items that we added to the standard COREQ checklist, ‘local relevance of the research question’ was reported in 100%, ‘international ethics statement was included’ in 99%, and ‘local ethics statement’ in 98%.

The five most common not reported items included: information on whether participants provided feedback on the research findings (96% not reported, item 28); repeat interviews were performed (85% not reported, item 18); participants refused (72% not reported, item 13); gender of the researcher (58% not reported, item 4); and relationship was established prior to study commencement (55% not reported, item 6). In terms of items that were not applicable, type of software (item 27) was not applicable in 61 publications, since data analysis was conducted manually. ‘Local ethics statement’ was not applicable in one publication, which was conducted in undocumented migrants in Serbia where the political climate (regarding migrants) was not in favor of the research topic and findings.

## 4. Discussion

This study assessed the characteristics and quality of reporting involving the largest dataset of qualitative and mixed-methods publications emerging from a capacity building program in LMICs. The remit involved a wide range of countries (18), journals (32) and research themes (13). LMIC authors led on 96% of publications, about half of all first authors were female, and each publication had on average, five LMIC institutions represented in the authorship. Additionally, the study demonstrated that almost 90% of publications were graded as ‘good to excellent’ in terms of reporting quality. These findings highlight the vital role played by SORT IT in ensuring reporting quality, while strongly promoting gender equity and LMIC research leadership.

This study is important to both operational research scientists and those from academia who are involved with research training initiatives. It shows that publications from SORT IT are well reported and this can be replicated by others involved with similar research capacity building initiatives. It provides evidence that researchers and institutions can apply the COREQ checklist for ensuring adherence and accountability to reporting standards in operational research [22]. It also provides a useful baseline for future comparisons while SORT IT is being franchised to partner institutions, many of whom are independently implementing the SORT IT brand. Finally, we adapted the COREQ checklist to include important items such as ethics and research relevance and this improved tool can now be applied by others who plan to conduct similar evaluations. As in all fields of research, poorly designed, conducted or reported studies can lead to inappropriate findings and interpretation [22]. Use of the COREQ checklist during protocol development and manuscript writing can contribute to ensuring higher levels of evidence in the conduct and reporting of qualitative components in operational research [22].

The study strengths are that we included all qualitative and mixed-methods publications generated over a period of seven years; the methodology of scoring was pilot tested and a system for cross-validation of scores was in place; and data on authorship and equity aspects were sourced from a SORT IT database that is routinely validated during quarterly reporting of SORT IT performance targets.

The main study limitation was that we applied the COREQ checklist for qualitative components of mixed-methods publications, where due to word count limits prescribed by the 17 journals, reporting of the qualitative component was less exhaustive than with qualitative studies. Such word count limits could have hampered authors from including detailed information on various items in the COREQ checklist, and this in turn could have negatively influenced reporting completeness. A way forward is for operational research journals to be flexible on word count limits for mixed-method designs. Another limitation is that we did not assess the quantitative component of mixed-method studies. A limitation of focusing on the SORT IT dataset is that the drafting of study protocols and manuscripts follows a modus operandi that is rather standardized and in line with the COREQ requirements. As such, reporting quality is likely to be good, compared to other publication datasets where methodology and reporting rigor are less strong.

The study findings have a number of policy and practice implications. First, the proportion of publications having a cumulative score of ‘good to excellent’ was 89%, and this exceeds that in the literature. In a study by Godinho et al. involving 246 qualitative public health research publications from India, completeness of reporting was 43–57% (‘fair to poor’ reporting) [21]. In another study by Walsh et al. [20], involving 197 publications in nursing social science, quality of reporting was either graded ‘moderate’ (57%) or poor (38%). Several other qualitative studies from dentistry, nursing, organ transplant, and smoking practices showed a 40–60% completeness of reporting [15,16,17,18,19]. This begs the question: why is the quality of reporting substantially higher in SORT IT publications? The reasons are intuitive and include: embedding the use of the COREQ checklist into drafting of study protocols and writing manuscripts; rigorous hands-on mentorship by experienced mentors and critical appraisals; the presence of experts in qualitative research during trainings; and the inclusion of the COREQ checklist as a standard indicator of SORT IT trainings and performance standards. These measures can be replicated to improve reporting of qualitative research studies.

Second, strong representation of LMICs in research leadership by prominent (96%) first authorship and engaging with LMIC institutions in collaborative partnerships is noteworthy as it is fundamentally about promoting ‘local research for local solutions with local ownership’. The high proportion of first-author positions from LMICs is in stark contrast to what has been reported in the literature. For example, in a study of authorship by Iyer et al. involving 236 publications in the Lancet Global Health on research in LMICs, only 35% of the authors were affiliated with, or came from, LMICs [23]. The first author position should be considered a proxy of research leadership, and is an indicator of the success of research capacity-building in LMICs. An average of five LMIC institutions represented on each publication in this study is also an indicator of successful LMIC-LMIC collaborations achieved through the SORT IT partnership [24].

Third, although not included in the standard COREQ checklist, we believe that ethics reporting, both local and international, is indispensable for good-quality operational research and we strongly advocate for its inclusion in the COREQ checklist [25]. Reassuringly, 98% of SORT IT publications mentioned local ethics and 99% mentioned international ethics. We also verified if the local relevance of research was mentioned as this is an important indicator of ‘homegrown’ research. Ensuring that research is locally relevant is important to those expected to use the results for influencing policy and/or practice. The words of John Walley et al. summarizes this well, “if you want to get research into practice, first get practice into research” [2].

There are a number of items that should be better reported in future SORT IT studies such as: participant cross-checking which involves seeking feedback from research participants on the research findings. This participant validation can be achieved by de-briefing at the end of the interview, transcript return, or sharing consolidated findings. The choice depends on the feasibility. Considering the need for consensus in qualitative results, sharing of consolidated findings with participants would bring more acceptance and trustworthiness to the process. Other items that need improved reporting include: information on whether repeat interviews were performed; information on how many participants refused interviews; and reporting the gender of the researcher.

The median publication time was 14 months and this is rather long. Accelerating both the journal editorial and peer-review processes have been shown to considerably reduce the submission to publication time to less than three months in recent SORT IT studies [26]. This impetus needs to be applied to qualitative research.

Finally, the availability of altimetric data on the journal websites (such as views, citations and article downloads) in both open access and subscription journals was far from desirable with only 28% of publications providing open access to such data. We strongly advocate that all journals provide readers with altimetric data as this is a measure of research utility. Commendable examples that provide such data include the MDPI, PloS and BMC journals.

## 5. Conclusions

This study showcases the vital role that SORT IT has played in generating high-quality evidence in qualitative and mixed-methods operational research that can be used for informed decision making, while promoting LMIC research leadership and partnerships. The study will also serve as a baseline for ensuring quality control of publications while the SORT IT model is franchised in efforts to improve public health and achieve universal health coverage.

## Figures and Tables

**Table 1:** Characteristics of published operational research with a qualitative component (mixed-methods or qualitative study design) from the Structured Operational Research and Training Initiative courses (January 2015–July 2021).

Characteristics of Published Papers	N	(%)
**Total publications**	67	
**Number of countries where research was done**	18	
**Number of journals**	32	
**Journal impact factor, mean (range)**	2.5	(0.8–4.9)
**Research themes**		
Tuberculosis	30	(45)
HIV/AIDS	10	(15)
Other Infectious diseases	6	(9)
Migrant health	5	(7)
Tobacco	4	(6)
Non communicable diseases	4	(6)
Maternal and child health	2	(3)
Others ^1^	6	(9)
**LMIC author**		
First author	64	(96)
Corresponding author	63	(94)
Last author	55	(82)
**Gender equity**		
Female first author	30	(45)
**Affiliation of the first author ^2^**		
Academic institutions	30	(45)
International or national NGOs	21	(31)
Disease control programs/Ministries of health	16	(24)
**Institutions**		
Mean number from HICs per publication (range)	1	(0–5)
Mean number from LMICs per publication (range)	5	(1–11)
**Journal access type**		
Open access	63	(94)
Subscription-based	4	(6)

^1^ One each on antimicrobial resistance, internet addiction, mental health, alternative medicine, adolescent health, health systems. ^2^ A first author may have multiple affiliations.

**Table 2:** Quality of reporting of published operational research with a qualitative component (mixed-methods or qualitative study design) from the Structured Operational Research and Training Initiative courses (January 2015–July 2021) using the adapted COREQ checklist.

Quality of Reporting	N	(%)
**Total Publications**	67	
**COREQ scores**		
>80% (Excellent)	19	(28)
70–79% (Very good)	24	(36)
60–69% (Good)	17	(25)
50–59% (Fair)	5	(8)
<50% (Poor)	2	(3)

**Table 3:** Percentage of items in the adapted COREQ checklist which were reported in 67 published operational research studies (55 mixed methods and 12 qualitative designs) from the Structured Operational Research and Training Initiative courses (January 2015–July 2021).

Item No	Item Description	Not Applicable	Reported	
		n	n	(%) *
**Domain 1. Research team and reflexivity**				
1. Interviewer/facilitator	Which author/s conducted the interview or focus group?	0	67	(100)
2. Credentials	What were the researcher’s credentials? e.g., PhD, MD	0	39	(58)
3. Occupation	What was their occupation at the time of the study?	0	38	(57)
4. Gender	Was the researcher male or female?	0	28	(42)
5. Experience and training	What experience or training did the researcher have?	0	55	(82)
6. Relationship established	Was a relationship established prior to study commencement?	0	30	(45)
7. Participant knowledge of the interviewer	What did the participants know about the researcher?, e.g., personal goals, reasons for doing the research	0	65	(97)
8. Interviewer characteristics	What characteristics were reported about the interviewer/facilitator?, e.g., Bias, assumptions, reasons and interests in the research topic	0	31	(46)
**Domain 2. Study design**				
9. Methodological orientation and theory	What methodological orientation was stated to underpin the study?, e.g., grounded theory, discourse analysis, ethnography, phenomenology, content analysis	0	66	(99)
10. Sampling	How were participants selected?, e.g., purposive, convenience, consecutive, snowball	0	66	(99)
11. Method of approach	How were participants approached?, e.g., face-to-face, telephone, mail, email	0	61	(91)
12. Sample size	How many participants were in the study?	0	67	(100)
13. Non-participation	How many people refused to participate or dropped out? Reasons?	0	19	(28)
14. Setting of data collection	Where was the data collected?, e.g., home, clinic, workplace	1	57	(86)
15. Presence of non-participants	Was anyone else present besides the participants and researchers?	1	39	(59)
16. Description of sample	What are the important characteristics of the sample?, e.g., demographic data, date	0	35	(52)
17. Interview guide	Were questions, prompts, guides provided by the authors? Was it pilot tested?	0	41	(61)
18. Repeat interviews	Were repeat interviews carried out? If yes, how many?	0	10	(15)
19. Audio/visual recording	Did the research use audio or visual recording to collect the data?	0	62	(93)
20. Field notes	Were field notes made during and/or after the interview or focus group?	0	45	(67)
21. Duration	What was the duration of the interviews or focus group?	0	48	(72)
22. Data saturation	Was data saturation discussed?	0	37	(55)
23. Transcripts returned	Were transcripts returned to participants for comment and/or correction?	0	39	(58)
**Domain 3. Analysis and reporting**				
24. Number of data coders	How many data coders coded the data?	0	62	(93)
25. Description of the coding tree	Did authors provide a description of the coding tree?	0	46	(69)
26. Derivation of themes	Were themes identified in advance or derived from the data?	0	65	(97)
27. Software	What software, if applicable, was used to manage the data?	61	6	(100)
28. Participant checking	Did participants provide feedback on the findings?	0	3	(4)
29. Quotations presented	Were participant quotations presented to illustrate the themes / findings? Was each quotation identified?, e.g., participant number	0	65	(97)
30. Data and findings consistent	Was there consistency between the data presented and the findings?	0	66	(99)
31. Clarity of major themes	Were major themes clearly presented in the findings?	0	66	(99)
32. Clarity of minor themes	Is there a description of diverse cases or discussion of minor themes?	0	64	(96)
**Other information**				
33. Local relevance of the research question	Indicated/mentioned in the paper	0	67	(100)
34. Local ethics statement	Indicated/mentioned in the paper	1	65	(98)
35. International ethics statement	Indicated/mentioned in the paper	0	66	(99)

* Percentages are calculated using the total number of applicable items as the denominator (maximum number of items = 35), where the total number of applicable items is 35 minus the number of non-applicable items.

## Data Availability

Requests to access these data should be sent to the corresponding author.

## Supplementary Materials

The following supporting information can be downloaded at: https://www.mdpi.com/article/10.3390/tropicalmed7100305/s1, Supplementary File S1: List of 67 publications included in this study; Supplementary File S2: COREQ—adapted checklist with 35 items. Items 33, 34, and 35 in red were added by TDR.

## Open Access Statement and Disclaimer

In accordance with WHO’s open access publication policy for all work funded by WHO or authored/co-authored by WHO staff members, WHO retains the copyright of this publication through a Creative Commons Attribution IGO license (http://creativecommons.org/licenses/by/4.0/igo/legalcode (last accessed on 23 February 2022)) which permits unrestricted use, distribution and reproduction in any medium provided the original work is properly cited. There should be no suggestion that WHO endorses any specific organization, products, or services. The views expressed in this article are those of the authors and do not necessarily reflect those of their affiliated institutions. The use of the WHO logo is not permitted. This notice should be preserved along with the article’s original URL.
