# Supplementary material for: Quality, Equity and Partnerships in Mixed Methods and Qualitative Research during Seven Years of Implementing the Structured Operational Research and Training Initiative in 18 Countries
Source: Trop Med Infect Dis. 2022 Oct 17;7(10):305. doi: 10.3390/tropicalmed7100305 (PMC9610844; doi:10.3390/tropicalmed7100305)
Supplement: Supplementary file 1 [file tropicalmed-07-00305-s001.zip › Supplementary File S1.pdf]

Supplementary File S1. List of 67 publications included in the study.

1. Arsenijević, J.; Burtscher, D.; Ponthieu, A.; Severy, N.; Contenta, A.; Moissaing, S.; Argenziano, S.; Zamatto, F.; Zachariah, R.; Ali, E.; et al. “I feel like I am less than other people”: Health-related vulnerabilities of male migrants travelling alone on their journey to Europe. Soc. Sci. Med. 2018, 209, 86–94, doi:10.1016/j.socscimed.2018.05.038.
2. Oliveira, A.; Selvaraj, K.; Tripathy, J.P.; Betodkar, U.; Cacodcar, J.; Wadkar, A. Kyasanur forest disease vaccination coverage and its perceived barriers in Goa, India-A mixed methods operational research. PLoS One 2019, 14, 1–13, doi:10.1371/journal.pone.0226141.
3. Pulido Tarquino, I.A.; Venables, E.; de Amaral Fidelis, J.M.; Giuliani, R.; Decroo, T. “I take my pills every day, but then it goes up, goes down. I don’t know what’s going on”: Perceptions of HIV virological failure in a rural context in Mozambique. A qualitative research study. PLoS One 2019, 14, 1–13, doi:10.1371/journal.pone.0218364.
4. Raghuveer, P.; Anand, T.; Tripathy, J.P.; Nirgude, A.S.; Reddy, M.M.; Nandy, S.; Shaira, H.; Naik, P.R. Opportunistic screening for diabetes mellitus and hypertension in primary care settings in Karnataka, India: A few steps forward but still some way to go. F1000Research 2020, 9, doi:10.12688/f1000research.22825.1.
5. Singh, A.R.; Kharate, A.; Bhat, P.; Kokane, A.M.; Bali, S.; Sahu, S.; Verma, M.; Nagar, M.; Kumar, A.M.V. Isoniazid preventive therapy among children living with tuberculosis patients: Is it working? A mixed-method study from Bhopal, India. J. Trop. Pediatr. 2017, 63, 274–285, doi:10.1093/tropej/fmw086.
6. Prasanna, T.; Jeyashree, K.; Chinnakali, P.; Bahurupi, Y.; Vasudevan, K.; Das, M. Catastrophic costs of tuberculosis care: a mixed methods study from Puducherry, India. Glob. Health Action 2018, 11, doi:10.1080/16549716.2018.1477493.
7. Tenzin, K.; Dorji, T.; Choeda, T.; Wangdi, P.; Oo, M.M.; Tripathy, J.P.; Tenzin, T.; Tobgay, T. Internet addiction among secondary school adolescents: A mixed methods study. J. Nepal Med. Assoc. 2019, 57, 344–351, doi:10.31729/jnma.4292.
8. Sande, O.; Burtscher, D.; Kathumba, D.; Tweya, H.; Phiri, S.; Gugsa, S. Patient and nurse perspectives of a nurse-led community-based model of HIV care delivery in Malawi: A qualitative study. BMC Public Health 2020, 20, 1–8, doi:10.1186/s12889-020-08721-6.
9. Gamariel, F.; Isaakidis, P.; Tarquino, I.A.P.; Beirão, J.C.; O’Connell, L.; Mulieca, N.; Gatoma, H.P.; Cumbe, V.F.J.; Venables, E. Access to health services for men who have sex with men and transgender women in Beira, Mozambique: A qualitative study. PLoS One 2020, 15, 1–16, doi:10.1371/journal.pone.0228307.
10. Muthuvel, T.; Govindarajulu, S.; Isaakidis, P.; Shewade, H.D.; Rokade, V.; Singh, R.; Kamble, S. “I Wasted 3 Years, Thinking It’s Not a Problem”: Patient and Health System Delays in Diagnosis of Leprosy in India: A Mixed-Methods Study. PLoS Negl. Trop. Dis. 2017, 11, 1–15, doi:10.1371/journal.pntd.0005192.

11. Veeraiah, S.; Elangovan, V.; Tripathy, J.P.; Krishnamurthy, A.; Anand, T.; Reddy, M.M.; Sudhakar, R.; Niraimathi, K.; Subramani, D.; Rajaraman, S.; et al. Quit attempts among tobacco users identified in the Tamil Nadu Tobacco Survey of 2015/2016: A 3 year follow-up mixed methods study. BMJ Open 2020, 10, doi:10.1136/bmjopen-2019-034607.
12. Kanakaraju, M.; Nagaraja, S.B.; Satyanarayana, S.; Babu, Y.R.; Madhukeshwar, A.K.; Narasimhaiah, S. Chest Radiography and Xpert MTB/RIF® Testing in Persons with Presumptive Pulmonary TB: Gaps and Challenges from a District in Karnataka, India. Tuberc. Res. Treat. 2020, 2020, 1–10, doi:10.1155/2020/5632810.
13. Lin Aung, N.H.H.; Soe, K.T.; Kumar, A.M.V.; Saw, S.; Aung, S.T. What are the barriers for uptake of antiretroviral therapy in HIV-infected tuberculosis patients? A mixed-methods study from Ayeyawady region, Myanmar. Trop. Med. Infect. Dis. 2020, 5, 1–15, doi:10.3390/tropicalmed5010041.
14. Dey, A.; Thekkur, P.; Ghosh, A.; Dasgupta, T.; Bandopadhyay, S.; Lahiri, A.; Sanju, C.S.V.; Dinda, M.K.; Sharma, V.; Dimari, N.; et al. Active case finding for tuberculosis through TOUCH agents in selected high TB burdenwards of Kolkata, India: A mixed methods study on outcomes and implementation challenges. Trop. Med. Infect. Dis. 2019, 4, doi:10.3390/tropicalmed4040134.
15. Krishnamurthy, S.K.G.; Nagaraja, S.B.; Anand, T.; Sagili, K.D.; Gowda, C.; S.; Poojar, B.; Satyanarayana, S. Threefold Increase in the Number of Drug Resistant TB Cases after Introduction of Universal Drug Susceptibility Testing: Experiences from Two South India Districts. J. Tuberc. Res. 2020, 08, 42–52, doi:10.4236/jtr.2020.82005.
16. Kumar, A.; Bhagabaty, S.M.; Tripathy, J.P.; Selvaraj, K.; Purkayastha, J.; Singh, R. Delays in diagnosis and treatment of breast cancer and the pathways of care: A mixed methods study from a tertiary cancer centre in north east India. Asian Pacific J. Cancer Prev. 2019, 20, 3711–3721, doi:10.31557/APJCP.2019.20.12.3711.
17. Prabhu, A.; Agarwal, U.; Tripathy, J.P.; Singla, N.; Sagili, K.; Thekkur, P.; Sarin, R. "99DOTS"techno-supervision for tuberculosis treatment – A boon or a bane? Exploring challenges in its implementation at a tertiary centre in Delhi, India. Indian J. Tuberc. 2020, 67, 46–53, doi:10.1016/j.ijtb.2019.08.010.
18. Mahalakshmy, T.; Premarajan, K.C.; Soundappan, K.; Rajarethinam, K.; Krishnamoorthy, Y.; Rajalatchumi, A.; Mathavaswami, V.; Chandar, D.; Chinnakali, P.; Dongre, A.R. A Mixed Methods Evaluation of Adolescent Friendly Health Clinic Under National Adolescent Health Program, Puducherry, India. Indian J. Pediatr. 2019, 86, 132–139, doi:10.1007/s12098-018-2755-4.
19. Hein, K.T.; Maung, T.M.; Htet, K.K.K.; Shewade, H.D.; Tripathy, J.P.; Oo, S.M.; Lin, Z.; Thi, A. Low uptake of malaria testing within 24 h of fever despite appropriate health-seeking among migrants in Myanmar: A mixed-methods study. Malar. J. 2018, 17, 1–13, doi:10.1186/s12936-018-2546-4.

20. Aye, L.L.; Tripathy, J.P.; Maung, T.M.; Oo, M.M.; Nwe, M.L.; Thu, H.M.M.; Ko, K.; Kaung, K.K. Experiences from the pilot implementation of the Package of Essential Non-communicable Disease Interventions (PEN) in Myanmar, 2017-18: A mixed methods study. PLoS One 2020, 15, 1–19, doi:10.1371/journal.pone.0229081.
21. Vijayageetha, M.; Kumar, A.M.V.; Ramakrishnan, J.; Sarkar, S.; Papa, D.; Mehta, K.; Joseph, N.M.; Rajaram, M.; Rajaa, S.; Chinnakali, P. Tuberculosis screening among pregnant women attending a tertiary care hospital in Puducherry, South India: is it worth the effort? Glob. Health Action 2019, 12, doi:10.1080/16549716.2018.1564488.
22. Reddy, M.M.; Thekkur, P.; Ramya, N.; Kamath, P.B.T.; Shastri, S.G.; Kumar, R.B.N.; Chinnakali, P.; Nirgude, A.S.; Rangaraju, C.; Somashekar, N.; et al. To start or to complete?–Challenges in implementing tuberculosis preventive therapy among people living with HIV: a mixed-methods study from Karnataka, India. Glob. Health Action 2020, 13, doi:10.1080/16549716.2019.1704540.
23. Mehta, K.; Kumar, A.M.V.; Chawla, S.; Chavda, P.; Selvaraj, K.; Shringarpure, K.S.; Solanki, D.M.; Verma, P.B.; Rewari, B.B. 'M-TRACK' (mobile phone reminders and electronic tracking tool) cuts the risk of pre-treatment loss to follow-up by 80% among people living with HIV under programme settings: a mixed-methods study from Gujarat, India. Glob. Health Action 2018, 11, doi:10.1080/16549716.2018.1438239.
24. Frederick, A.; Das, M.; Mehta, K.; Kumar, G.; Satyanarayana, S. Pharmacy based surveillance for identifying missing tuberculosis cases: A mixed methods study from South India. Indian J. Tuberc. 2021, 68, 51–58, doi:10.1016/j.ijtb.2020.09.017.
25. Oo, T.; Kyaw, K.W.Y.; Soe, K.T.; Saw, S.; Satyanarayana, S.; Aung, S.T. Magnitude and reasons for pre-diagnosis attrition among presumptive multi-drug resistant tuberculosis patients in Bago Region, Myanmar: A mixed methods study. Sci. Rep. 2019, 9, 1–9, doi:10.1038/s41598-019-43562-3.
26. Farley, E.; Bala, H.M.; Lenglet, A.; Mehta, U.; Abubakar, N.; Samuel, J.; De Jong, A.; Bil, K.; Oluyide, B.; Fotso, A.; et al. "I treat it but i don't know what this disease is": A qualitative study on noma (cancrum oris) and traditional healing in northwest Nigeria. Int. Health 2019, 12, 28–35, doi:10.1093/inthealth/ihz066.
27. Zaw, M.K.K.; Satyanarayana, S.N.; Htet, K.K.K.; Than, K.K.; Aung, C.T. Is myanmar on the right track after declaring leprosy elimination? Trends in new leprosy cases (2004–2018) and reasons for delay in diagnosis. Lepr. Rev. 2020, 91, 25–40, doi:10.47276/lr.91.1.25.
28. Eleftherakos, C.; Van Den Boogaard, W.; Barry, D.; Severy, N.; Kotsioni, I.; Roland-Gosselin, L. "I prefer dying fast than dying slowly", how institutional abuse worsens the mental health of stranded Syrian, Afghan and Congolese migrants on Lesbos island following the implementation of EU-Turkey deal. Confl. Health 2018, 12, 1–11, doi:10.1186/s13031-018-0172-y.
29. Ismail, I.M.; Madhukeshwar, A.K.; Naik, P.R.; Nayarmoole, B.M.; Satyanarayana, S. Magnitude and reasons for gaps in tuberculosis diagnostic testing and treatment

initiation: An operational research study from Dakshina Kannada, South India. J. Epidemiol. Glob. Health 2020, 10, 326–336, doi:10.2991/jegh.k.200516.001.

30. Kumar, S.; Gupte, H.A.; Isaakidis, P.; Mishra, J.K.; Munjattu, J.F. “They don’t like us. . ..”: Barriers to antiretroviral and opioid substitution therapy among homeless HIV positive people who inject drugs in Delhi: A mixed method study. PLoS One 2018, 13, 1–14, doi:10.1371/journal.pone.0203262.
31. Wagh, A.N.; Mugudalabetta, S.; Gutierrez, N.O.; Padebettu, K.; Pandey, A.K.; Pandey, B.K.; Thulasingam, M.; Satyanarayana, S.; Dongre, A. Does appreciative inquiry decrease false positive diagnosis during leprosy case detection campaigns in Bihar, India? An operational research study. PLoS Negl. Trop. Dis. 2018, 12, 1–16, doi:10.1371/journal.pntd.0007004.
32. Rajan, J.C.; Anand, T.; Nagaraja, S.B.; Ulahannan, S.K.; Sagili, K.; Sarojini, M.M. Tuberculosis Treatment Completion for Tribal Patients in Kerala: Needs Constant Push! J. Tuberc. Res. 2019, 07, 185–201, doi:10.4236/jtr.2019.74018.
33. Jakasania, A.; Shringarpure, K.; Kapadia, D.; Sharma, R.; Mehta, K.; Prajapati, A.; Kathirvel, S. “Side effects--part of the package”: a mixed methods approach to study adverse events among patients being programmatically treated for DR-TB in Gujarat, India. BMC Infect. Dis. 2020, 20, 1–12, doi:10.1186/s12879-020-05660-w.
34. Sandar, W.P.; Saw, S.; Kumar, A.M.V.; Camara, B.S.; Sein, M.M. Wounds, antimicrobial resistance and challenges of implementing a surveillance system in myanmar: A mixed-methods study. Trop. Med. Infect. Dis. 2021, 6, doi:10.3390/tropicalmed6020080.
35. Linn, N.Y.Y.; Tripathy, J.P.; Maung, T.M.; Saw, K.K.; Maw, L.Y.W.; Thapa, B.; Lin, Z.; Thi, A. How are the village health volunteers deliver malaria testing and treatment services and what are the challenges they are facing? A mixed methods study in Myanmar. Trop. Med. Health 2018, 46, 1–14, doi:10.1186/s41182-018-0110-0.
36. Chavan, V.V.; Dalal, A.; Nagaraja, S.; Thekkur, P.; Mansoor, H.; Meneguim, A.; Paryani, R.; Singh, P.; Kalon, S.; Das, M.; et al. Ambulatory management of pre- And extensively drug resistant tuberculosis patients with imipenem delivered through port-a-cath: A mixed methods study on treatment outcomes and challenges. PLoS One 2020, 15, 1–18, doi:10.1371/journal.pone.0234651.
37. Snyman, L.; Venables, E.; Duran, L.T.; Mohr, E.; Azevedo, V.D.; Harmans, X.; Isaakidis, P. “I didn’t know so many people cared about me”: Support for patients who interrupt drug-resistant TB treatment. Int. J. Tuberc. Lung Dis. 2018, 22, 1023–1030, doi:10.5588/ijtld.17.0826.
38. Nirgude, A.S.; Kumar, A.M.V.; Collins, T.; Naik, P.R.; Parmar, M.; Tao, L.; Akshaya, K.M.; Raghuveer, P.; Yatnatti, S.K.; Nagendra, N.; et al. ‘I am on treatment since 5 months but I have not received any money’: coverage, delays and implementation challenges of ‘Direct Benefit Transfer’ for tuberculosis patients–a mixed-methods study from South India. Glob. Health Action 2019, 12, doi:10.1080/16549716.2019.1633725.

39. Hein, Z.N.M.; Maung, T.M.; Aung, P.P.; Mon, N.O.; Han, W.W.; Oo, T.; Linn, N.Y.Y.; Thi, A.; Wai, K.T. Do we need to go further to train healthcare providers in the targeted regions for malaria elimination in Myanmar? A mixed-methods study. Trop. Med. Health 2020, 48, 1–8, doi:10.1186/s41182-020-00196-w.
40. Akshaya, K.M.; Shewade, H.D.; Aslesh, O.P.; Nagaraja, S.B.; Nirgude, A.S.; Singarajipura, A.; Jacob, A.G. “Who has to do it at the end of the day? Programme officials or hospital authorities?” Airborne infection control at drug resistant tuberculosis (DR-TB) centres of Karnataka, India: A mixed-methods study. Antimicrob. Resist. Infect. Control 2017, 6, 1–10, doi:10.1186/s13756-017-0270-4.
41. Phyo, A.M.; Kumar, A.M.V.; Soe, K.T.; Kyaw, K.W.Y.; Thu, A.S.; Wai, P.P.; Aye, S.; Saw, S.; Maung, H.M.W.; Aung, S.T. Contact investigation of multidrug-resistant tuberculosis patients: A mixed-methods study from Myanmar. Trop. Med. Infect. Dis. 2020, 5, doi:10.3390/tropicalmed5010003.
42. Laxmeshwar, C.; Stewart, A.G.; Dalal, A.; Kumar, A.M.V.; Kalaiselvi, S.; Das, M.; Gawde, N.; Thi, S.S.; Isaakidis, P. Beyond ‘cure’ and ‘treatment success’: Quality of life of patients with multidrug-resistant tuberculosis. Int. J. Tuberc. Lung Dis. 2019, 23, 73–81, doi:10.5588/ijtld.18.0149.
43. Episkopou, M.; Venables, E.; Whitehouse, K.; Eleftherakos, C.; Zamatto, F.; De Bartolome Gisbert, F.; Severy, N.; Barry, D.; Van Den Bergh, R. In island containment: A qualitative exploration of social support systems among asylum seekers in a mental health care programme on Lesvos Island, Greece. Confl. Health 2019, 13, 1–14, doi:10.1186/s13031-019-0218-9.
44. Philip, S.; Isaakidis, P.; Sagili, K.D.; Meharunnisa, A.; Mrithyunjayan, S.; Kumar, A.M.V. “They know, they agree, but they don’t do” - The paradox of tuberculosis case notification by private practitioners in Alappuzha district, Kerala, India. PLoS One 2015, 10, 1–13, doi:10.1371/journal.pone.0123286.
45. Anand, T.; Kishore, J.; Isaakidis, P.; Gupte, H.A.; Kaur, G.; Kumari, S.; Jha, D.; Grover, S. Integrating screening for non-communicable diseases and their risk factors in routine tuberculosis care in Delhi, India: A mixed-methods study. PLoS One 2018, 13, 1–16, doi:10.1371/journal.pone.0202256.
46. Gummidi, B.; John, R.; Burugina Nagaraja, S.; Tripathy, J.P. Qualitative enquiry on irregular intake of antihypertensive medications to inform a model of care to improve blood pressure control. Contemp. Nurse 2020, 56, 455–465, doi:10.1080/10376178.2020.1844577.
47. Navya, N.; Jeyashree, K.; Madhukeshwar, A.K.; Anand, T.; Nirgude, A.S.; Nayarmoole, B.M.; Isaakidis, P. Are they there yet? Linkage of patients with tuberculosis to services for tobacco cessation and alcohol abuse - A mixed methods study from Karnataka, India. BMC Health Serv. Res. 2019, 19, 1–12, doi:10.1186/s12913-019-3913-8.

48. Spissu, C.; De Maio, G.; Van den Bergh, R.; Ali, E.; Venables, E.; Burtscher, D.; Ponthieu, A.; Ronchetti, M.; Mostarda, N.; Zamatto, F. It never happened to me, so I don't know if there are procedures": identification and case management of torture survivors in the reception and public health system of Rome, Italy. Torture J. 2018, 28, 38–55, doi:10.7146/torture.v28i2.106921.
49. Dorji, T.; Das, M.; Van Den Bergh, R.; Oo, M.M.; Gyamtsho, S.; Tenzin, K.; Tshomo, T.; Ugen, S. "if we miss this chance, it's futile later on" - Late antenatal booking and its determinants in Bhutan: A mixed-methods study. BMC Pregnancy Childbirth 2019, 19, 1–13, doi:10.1186/s12884-019-2308-5.
50. Thinn, K.K.; Thekkur, P.; Kyaw, N.T.T.; Aye, N.S.; Zaw, T.M.; Soan, P.; Hone, S.; Oo, H.N. Uptake of routine viral load testing among people living with HIV and its implementation challenges in Yangon region of Myanmar: A mixed-methods study. BMJ Open 2019, 9, 1–10, doi:10.1136/bmjopen-2019-032678.
51. Garg, T.; Gupta, V.; Sen, D.; Verma, M.; Brouwer, M.; Mishra, R.; Bhardwaj, M. Prediagnostic loss to follow-up in an active case finding tuberculosis programme: A mixed-methods study from rural Bihar, India. BMJ Open 2020, 10, 1–11, doi:10.1136/bmjopen-2019-033706.
52. Linn, S.Y.; Maung, T.M.; Tripathy, J.P.; Shewade, H.D.; Oo, S.M.; Linn, Z.; Thi, A. Barriers in distribution, ownership and utilization of insecticide-treated mosquito nets among migrant population in Myanmar, 2016: A mixed methods study. Malar. J. 2019, 18, 1–16, doi:10.1186/s12936-019-2800-4.
53. Siddaiah, A.; Ahmed, M.N.; Kumar, A.M.V.; D'Souza, G.; Wilkinson, E.; Maung, T.M.; Rodrigues, R. Tuberculosis notification in a private tertiary care teaching hospital in South India: A mixed-methods study. BMJ Open 2019, 9, 1–13, doi:10.1136/bmjopen-2018-023910.
54. Gowthamghosh, B.; Huidrom, R.; Arumugam, V.; Pathak, N.; Purohit, N.; Shewade, H.D.; Khanna, A.; Naik, P.R. Implementation of social protection schemes for people living with HIV in three districts of Rajasthan state, India – a mixed methods study. F1000Research 2020, 9, 248, doi:10.12688/f1000research.22285.1.
55. Gupte, H.A.; Zachariah, R.; Sagili, K.D.; Thawal, V.; Chaudhuri, L.; Verma, H.; Dongre, A.; Malekar, A.; Rigotti, N.A. Integration of tobacco cessation and tuberculosis management by NGOs in urban India: a mixed-methods study. Public Heal. Action 2019, 8, 50–58, doi:10.5588/PHA.17.0085.
56. Newtonraj, A.; Venables, E.; Selvaraj, K.; Kundu, D.; Purty, A.J.; Manikandan, M.; Shewade, H.D. Xpert negative means no TB: A mixed-methods study into early implementation of Xpert in Puducherry, India. J. Fam. Med. Prim. Care 2019, 8, 1379, doi:10.4103/JFMPC.JFMPC_421_18.
57. Verma, H.; Sagili, K.D.; Zachariah, R.; Aggarwal, A.; Dongre, A.; Gupte, H. Do incentivised community workers in informal settlements influence maternal and infant health in urban India? Public Heal. Action 2017, 7, 61–66, doi:10.5588/PHA.16.0056.

58. Shewade, H.D.; Govindarajan, S.; Sharath, B.N.; Tripathy, J.P.; Chinnakali, P.; Kumar, A.M.V.; Muthaiah, M.; Vivekananda, K.; Paulraj, A.K.; Roy, G. MDR-TB screening in a setting with molecular diagnostic techniques: Who got tested, who didn't and why? Public Heal. Action 2015, 5, 132–139, doi:10.5588/PHA.14.0098.
59. K. Selvaraj, A. M. V. Kumar, , S. Chawla, K. S. Shringarpure, P. Thekkur, C. Palanivel, P. B. Verma, A. N. Shah, K. N. Pandya, G. Roy, Z. Singh, B. B. Rewari, A.R.D. Are partners of HIV-infected people being tested for HIV? A mixed-methods research from Gujarat, India. Public Heal. Action 2017, 7, 46–54.
60. N. Ahuja, J. Kathiresan, T. Anand, P. Isaakidis, D.B. I have heard about it for the first time from you! Implementation of tobacco control law by police personnel in India. Public Heal. action 2018, 8, 194–201.
61. Chowdhury, S.; Chakraborty, P. pratim Universal health coverage - There is more to it than meets the eye. J. Fam. Med. Prim. Care 2017, 6, 169–170, doi:10.4103/jfmpc.jfmpc.
62. Patel, B.H.; Jeyashree, K.; Chinnakali, P.; Vijayageetha, M.; Mehta, K.G.; Modi, B.; Chavda, P.D.; Dave, P. V.; Zala, C.C.; Shewade, H.D.; et al. Cash transfer scheme for people with tuberculosis treated by the National TB Programme in Western India: A mixed methods study. BMJ Open 2019, 9, 1–12, doi:10.1136/bmjopen-2019-033158.
63. Shilpa D.M., Poonam Ramesh Naik, Hemant Deepak Shewade, H.S. Assessing the implementation of a mobile App-based electronic health record: A mixed-method study from South India. J Edu Heal. Promot 2020, 102, 1–6, doi:10.4103/jehp.jehp.
64. S. Chawla, K. Shringarpure, B. Modi, R. Sharma, B. B. Rewari, A. N. Shah, P. B. Verma, A. R. Dongre, A.M.V.K. Why are HIV-infected people not started on antiretroviral therapy? A mixed-methods study from Gujarat, India. Public Heal. Action 2017, 7, Syzdykova, A., Zolfo, M., Malta, A., Diro, E., O.
65. Shringarpure, K.S.; Isaakidis, P.; Sagili, K.D.; Baxi, R.K.; Das, M.; Daftary, A. "When treatment is more challenging than the disease": A qualitative study of MDR-TB patient retention. PLoS One 2016, 11, 1–12, doi:10.1371/journal.pone.0150849.
66. Thekkur, P.; Kumar, A.N.V.; Chinnakali, P.; Selvaraju, S.; Bairy, R.; Singh, A.R.; Nirgude, A.; Selvaraj, K.; Venugopal, V.; Shastri, S. Outcomes and implementation challenges of using daily treatment regimens with an innovative adherence support tool among HIV-infected tuberculosis patients in Karnataka, India: a mixed-methods study. Glob. Health Action 2019, 12, doi:10.1080/16549716.2019.1568826.
67. Kumar, A.M. V Tuberculosis-diabetes screening: how well are we doing? A mixed-methods study from North India. Public Heal. Action 2017, 7, Syzdykova, A., Zolfo, M., Malta, A., Diro, E., O.
