# Supplementary material for: Quality, Equity and Partnerships in Mixed Methods and Qualitative Research during Seven Years of Implementing the Structured Operational Research and Training Initiative in 18 Countries
Source: Trop Med Infect Dis. 2022 Oct 17;7(10):305. doi: 10.3390/tropicalmed7100305 (PMC9610844; doi:10.3390/tropicalmed7100305)
Supplement: Supplementary file 1 [file tropicalmed-07-00305-s001.zip › Supplementary File S2.pdf]

Supplementary fileS2. Box 1. COREQ —adapted checklist with 35 items. Items 33, 34, and 35 in red were added by TDR.

Journal publication ID

Reviewer Name:

	**Item No**	**Descriptions/Guiding questions**	**Reported Yes/No**	**Reported on Page No.**
**Domain 1. Research team and reflexivity**				
Personal characteristics				
Interviewer/facilitator	1	Which author/s conducted the interview or focus group?		
Credentials	2	What were the researcher's credentials? E.g. PhD, MD		
Occupation	3	What was their occupation at the time of the study?		
Gender	4	Was the researcher male or female?		
Experience and training	5	What experience or training did the researcher have?		
Relationship with participants				
Relationship established	6	Was a relationship established prior to study commencement?		
Participant knowledge of the interviewer	7	What did the participants know about the researcher? e.g. personal goals, reasons for doing the research		
Interviewer characteristics	8	What characteristics were reported about the interviewer/facilitator? e.g. Bias, assumptions, reasons and interests in the research topic		
**Domain 2. Study design**				

Theoretical framework				
Methodological orientation and theory	9	What methodological orientation was stated to underpin the study? e.g. grounded theory, discourse analysis, ethnography, phenomenology, content analysis		
Participant selection				
Sampling	10	How were participants selected? e.g. purposive, convenience, consecutive, snowball		
Method of approach	11	How were participants approached? e.g. face-to-face, telephone, mail, email		
Sample size	12	How many participants were in the study?		
Non-participation	13	How many people refused to participate or dropped out? Reasons?		
Setting				
Setting of data collection	14	Where was the data collected? e.g. home, clinic, workplace		
Presence of non-participants	15	Was anyone else present besides the participants and researchers?		
Description of sample	16	What are the important characteristics of the sample? e.g. demographic data, date		
Data collection				
Interview guide	17	Were questions, prompts, guides provided by the authors? Was it pilot tested?		
Repeat interviews	18	Were repeat interviews carried out? If yes, how many?		
Audio/visual recording	19	Did the research use audio or visual recording to collect the data?		

Field notes	20	Were field notes made during and/or after the interview or focus group?		
Duration	21	What was the duration of the interviews or focus group?		
Data saturation	22	Was data saturation discussed?		
Transcripts returned	23	Were transcripts returned to participants for comment and/or correction?		
**Domain 3. Analysis and reporting**				
Data analysis				
Number of data coders	24	How many data coders coded the data?		
Description of the coding tree	25	Did authors provide a description of the coding tree?		
Derivation of themes	26	Were themes identified in advance or derived from the data?		
Software	27	What software, if applicable, was used to manage the data?		
Participant checking	28	Did participants provide feedback on the findings?		
Reporting				
Quotations presented	29	Were participant quotations presented to illustrate the themes / findings? Was each quotation identified? e.g. participant number		
Data and findings consistent	30	Was there consistency between the data presented and the findings?		
Clarity of major themes	31	Were major themes clearly presented in the findings?		
Clarity of minor themes	32	Is there a description of diverse cases or discussion of minor themes?		
**Other information**				

Local relevance of the research question	33	Indicated/mentioned in the paper		
Local ethics statement included	34	Indicated/mentioned in the paper		
International ethics statement included	35	Indicated/mentioned in the paper		
